# Molecular Aspects of *H. pylori*-Related MALT Lymphoma

**DOI:** 10.4061/2011/193149

**Published:** 2011-01-24

**Authors:** Scott R. Owens, Lauren B. Smith

**Affiliations:** Department of Pathology, The University of Michigan, 1301 Catherine Rd., Room M5224 Medical Science I, Ann Arbor, MI 48109, USA

## Abstract

*Helicobacter pylori*-related extranodal marginal zone lymphoma of mucosa-associated lymphoid tissue is a paradigm for malignancy arising in an inflammatory background. While the diagnosis of *H. pylori* gastritis is often straightforward, distinction between severe gastritis and early lymphoma can be difficult and requires careful assessment of clinical findings in addition to histological features and immunohistochemical results. A number of cytogenetic abnormalities have been discovered in *H. pylori*-related lymphomas and several have clinical importance, related to the responsiveness of lymphoma to *H. pylori* eradication therapy, but routine molecular studies are not widely utilized. While molecular methods may be used in equivocal cases, a trial of conservative therapy is warranted given the propensity for these lymphomas to regress with eradication of the organism. Once therapy is initiated, care must be taken to avoid a premature assignment of disease refractoriness because complete response can take several months to more than a year. Cases truly refractory to *H. pylori* eradication therapy may be treated with adjuvant chemoradiation with a high response rate.

## 1. Introduction

**Figure 1:**
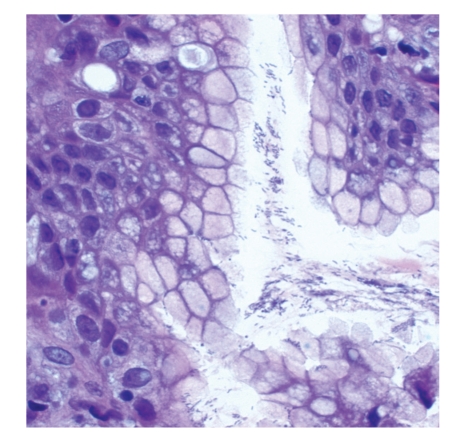
*Helicobacter pylori*, the underlying cause of most cases of gastric MALT lymphoma. The curved bacteria are seen in the surface mucus layer of the gastric pits and have a characteristic, curvilinear, “gull-wing” appearance (600x original magnification).

*Helicobacter pylori *is a common pathogen and the most frequent cause of gastric and duodenal ulcers (Figure 1) [1, 2]. This Gram-negative, curved bacillus was first recognized as the cause of human disease by Marshall and Warren in the 1980s and has since been classified as a class I carcinogen, potentially leading to gastric adenocarcinoma and, more commonly, extranodal marginal zone lymphoma of mucosa-associated lymphoid tissue (MALT lymphoma) [2–6]. Most *H. pylori*-related MALT lymphomas arise in the stomach, but extragastric lymphomas may also be related to the organism, particularly in the duodenum [7]. The gastritis caused by *H. pylori* is morphologically distinctive, with a band-like infiltrate of plasma cells and lymphocytes in the superficial gastric mucosa, typically in the antrum, accompanied by active (neutrophilic) inflammation in the mucus neck region of the mucosa, at the interface between the foveolae and glands. While *H. pylori* gastritis is most often easily recognized, the dividing line between severe gastritis and early MALT lymphoma is frequently indistinct [6, 7].

Several cytogenetic abnormalities have been described in gastrointestinal (GI) MALT lymphoma, and inappropriate activation of the NF-*κ*B pathway is a common thread [8–11]. While molecular assays for these abnormalities are available, guidelines for their routine use are not widely accepted, and the vast majority of cases of gastric as well as many extragastric, MALT lymphomas respond to conservative therapy aimed at eradication of infection [8, 12–17]. Some cytogenetic translocations seen in a subset of MALT lymphomas, however, are associated with resistance to antibiotic therapy. The routine use of molecular assays in diagnosis and prognosis of MALT lymphomas is controversial, and many cases are negative for any known cytogenetic abnormality [18–20].

This paper summarizes the current knowledge of cytogenetic abnormalities in GI MALT lymphoma, with particular attention to *H. pylori*-related gastric lymphomas. Currently available molecular testing methods are discussed, followed by a practical approach to their use in diagnosis. Finally, recommendations for disease followup are offered, with an emphasis on the utility of conservative therapy and avoidance of a premature determination of refractoriness to *H. pylori *eradication therapy.

## 2. MALT Lymphoma: A Review of Concepts

**Figure 2:**
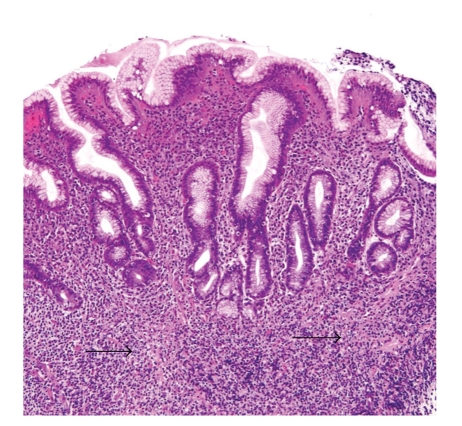
MALT lymphoma. The histomorphology in this case closely resembles severe *H. pylori* gastritis. Note the infiltration of the muscularis mucosae at the base of the mucosa (arrows). The clinical information was crucial in this case, as diffusely thickened gastric folds were seen endoscopically (400x original magnification).

MALT lymphoma (Figure 2) is a low-grade B-cell lymphoma composed predominantly of small lymphocytes, first described around the same time as *H. pylori* by Isaacson and Wright [21]. Morphologically, the lymphoma cells may be centrocyte-like (with small nuclei and scant cytoplasm, resembling follicle center cells) or monocytoid (with ample pale cytoplasm and indented nuclei), often with admixed centroblast-like cells (large cells that may have prominent nucleoli) [6]. Monocytoid cells are fairly characteristic of MALT lymphoma, but individual cases are commonly a mixture of the three cell types, with centroblast-like large cells typically being individually scattered. Plasmacytic differentiation is common, and some cases are almost completely plasmacytic in appearance [6, 22]. While immunohistochemistry (IHC) is useful in diagnosis, there remains no specific IHC marker for MALT lymphoma [7, 18].

MALT lymphoma accounts for about 8% of all B-cell lymphomas and tends to occur in older individuals with a nearly equal sex distribution [22]. About 85% of all GI MALT lymphomas occur in the stomach, and isolated involvement of the small intestine is rare [7]. Conversely, about 25% of gastric MALT lymphomas are accompanied by involvement of other GI sites, and there is evidence that it is a truly systemic disease [6, 18, 23]. The vast majority of GI MALT lymphomas are thought to be related to *H. pylori* infection, which is believed to lead to malignant transformation via chronic antigenic stimulation resulting in the clonal expansion of subpopulations of B cells [18, 24–27]. The noninfected stomach is largely devoid of lymphoid tissue; *H. pylori* gastritis owes its appearance to the acquisition of a lymphoplasmacytic infiltrate in the gastric lamina propria, which may or may not be accompanied by B-cell nodules and even germinal centers [6, 7, 18]. This type of tissue is termed “acquired MALT” to distinguish it from “native MALT” of the type seen, for example, in the distal ileum, where germinal centers make up the Peyer's patches.

Interestingly, while MALT lymphoma is a clonal B-cell neoplasm, the process of lymphomagenesis is believed to be driven by activated T cells [7, 27–29]. *H. pylori* strains expressing the *CagA* gene have been associated with significant morbidity, and this gene may play a role in lymphomagenesis [30]. The transformation of *H. pylori*-related MALT lymphoma to diffuse large B-cell lymphoma (DLBCL) has been postulated, although the exact mechanism of such a transformation remains obscure [6, 11, 18]. DLBCL is the most common GI lymphoma overall and the presence of a large cell component should be mentioned in the diagnosis when it occurs in association with MALT lymphomas [7, 22]. Sheets of large (centroblast-like) cells should prompt an unequivocal diagnosis of DLBCL, even when a recognizable low-grade MALT component is also present. Furthermore, such a low-grade component should be mentioned with the diagnosis of DLBCL when present. The former diagnosis of “high-grade MALT lymphoma” is no longer recognized and should not be used.

## 3. Cytogenetic Abnormalities in Gastrointestinal MALT Lymphoma

### 3.1. t(11;18)(q21;q21) API2/MALT1

The t(11;18) translocation involves the fusion of the N-terminus of *API2* (apoptosis inhibitor-2) on chromosome 11 and the C-terminus of *MALT1* (MALT lymphoma associated translocation) on chromosome 18. It is the most common translocation found in MALT lymphomas of the GI tract, involving up to 25% of gastric MALT lymphoma and 40%–60% of MALT lymphoma occurring in the small intestine [31, 32]. 

The presence of this translocation correlates with resistance to antibiotic therapy and MALT occurring without concomitant chronic active *H. pylori* gastritis, although it has been seen in specific strains of *H. pylori* infection [32, 33]. Cases with this translocation are more likely to have disseminated rather than stage I disease, but it is infrequently associated with diffuse large B-cell lymphoma [34, 35]. Some evidence suggests that plasmacytic morphology may not be seen in such cases [36]. 

Assays for the presence of t(11;18) are widely available. Methods include fluorescence in situ hybridization (FISH) or reverse transcription polymerase chain reaction (RT-PCR), both of which can be performed on fresh tissue or archival formalin-fixed paraffin-embedded tissue. FISH is more sensitive in the rare cases that do not involve the most common breakpoint. Dual color dual fusion FISH has been in use for the past ten years utilizing metaphase chromosomes or interphase nuclei [37]. In addition, break-apart MALT1 FISH probes are available, relatively easily interpreted in comparison to fusion probes, and may have the advantage of being useful in detecting MALT1 rearrangements in either t(11;18) or t(14;18) (see below) using one assay. IHC for BCL10 protein will show nuclear expression in many cases with the translocation; however, this is not specific and can be seen in t(1;14)(p22;q32) in association with the *BCL10/IGH* fusion [32]. The vast majority of cases with t(11;18) will show weak cytoplasmic MALT1 protein expression using IHC [38].

### 3.2. t(14;18)(q32;q21) IgH/MALT1

The t(14;18) translocation involves the immunoglobulin heavy chain gene (*IgH*) on chromosome 14 and *MALT1* on chromosome 18 which activates the NF-*κ*B pathway [39]. This translocation may coexist with trisomies (3, 12, or 18). While the breakpoint on chromosome 18 is in the same region as that seen in follicular lymphoma, the gene involved is *MALT1* rather than *BCL2*; rare cases of MALT lymphoma have reportedly been associated with the *IgH-BCL2* fusion [40]. While IgH/MALT1 is present in a large minority of MALT lymphomas, it occurs more commonly in non-GI sites. It has been reported, however, in unusual GI sites such as the liver [41].

Assays to detect t(14;18) include dual fusion FISH and PCR. IHC for MALT1 and BCL10 is highly sensitive, typically exhibiting strong, uniform cytoplasmic expression [38]; however, neither of these immunostains is specific for this translocation, as weak cytoplasmic staining for MALT1 and strong nuclear staining for BCL10 are characteristic of t(11;18). As these immunostains can be expressed in other translocations, they are not specific markers.

### 3.3. t(1;14)(p22;q32) BCL10/IGH

The t(1;14) translocation juxtaposes the *BCL10* gene on chromosome 1 with the *IgH* gene on chromosome 14. A variant translocation involving lambda light chain on chromosome 2 has also been reported [42]. These translocations are present in a minority of MALT lymphoma (1%–3%); however, it is important as gastric MALT lymphoma with this translocation can also demonstrate antibiotic resistance [43]. It has not been reported in other non-Hodgkin lymphomas. As mentioned previously, IHC for BCL10 shows strong nuclear expression. MALT1 is weakly expressed in the cytoplasm [38].

### 3.4. t(3;14)(q27;q32) BCL6/IGH and Other BCL6 Rearrangements

*BCL6* translocations are involved in a small number of MALT lymphomas, approximately 1%-2% in one large study [44]. The translocation can involve the immunoglobulin heavy chain, light chains, or other partners. Of relevance in GI cases, it has been reported in cases of diffuse large B-cell lymphoma with concurrent MALT in the stomach [45].

*BCL6* rearrangements can be identified by FISH using dual color break-apart probes. In a subset of *BCL6* rearranged cases, IHC for BCL6 protein will show nuclear staining of the lymphocytes which can cause diagnostic confusion with follicular lymphoma [44]. Only 25%–30% of the cases with the translocation in this study showed BCL6 expression by IHC. The number of cases is small, and additional large studies are needed.

### 3.5. t(3;14)(p14.1;q32) FOXP1/IGH

This translocation can be seen in diffuse large B-cell lymphoma and MALT lymphomas (ocular, thyroid, and cutaneous) without the t(11;18) [46]; it has not been commonly associated with MALT lymphomas of the GI tract.

### 3.6. Trisomy 3 and 18

Chromosomal trisomies are commonly detected in gastrointestinal marginal zone lymphomas but are nonspecific. Trisomy 3q27 is the most common chromosomal abnormality in gastrointestinal lymphomas. In one large series, it was present in 50%–65% of low-grade MALT lymphomas from stomach and small intestine [47]; however, other series showed a lower prevalence [31, 48]. It occurs in both low-grade marginal zone lymphomas and DLBCL and is more common in patients with higher stage disease [49]. Trisomy 18 can be seen independently or in association with trisomy 3 and has also been correlated with more aggressive disease, especially in gastrointestinal lymphomas classified as diffuse large B-cell lymphoma [50]. Both trisomies can be detected using FISH with chromosomal enumeration probes, and break-apart probes for BCL6 and MALT1 may also potentially identify these abnormalities.

## 4. Diagnosis of MALT Lymphoma and Use of Molecular Assays: A Pragmatic Approach

The distinction between severe *H. pylori* gastritis and early MALT lymphoma is often difficult and requires careful assessment of clinical findings as well as histomorphology and IHC. A carefully reasoned and evidence-based diagnosis of an incipient MALT lymphoma may not be clinically helpful when the lack of endoscopic findings makes it impossible for the gastroenterologist to find an appropriate site for rebiopsy to assess the effectiveness of therapy. Thus, discussion with clinical colleagues and a conscientious search of the clinical and endoscopic record for lesions including masses, malignant-appearing ulcers, and diffusely thickened gastric folds are helpful in evaluating the patient for malignancy. In reality, the vast majority of gastric MALT lymphomas—possibly as many as 80% or more—will regress in time with conservative *H. pylori* eradication therapy, including cases that are organism negative by IHC [51]. Some evidence suggests that MALT lymphomas in locations outside the stomach may also regress with conservative therapy. As a result, erring on the side of diagnostic caution in the setting of dubious clinical findings is prudent.

**Figure 3:**
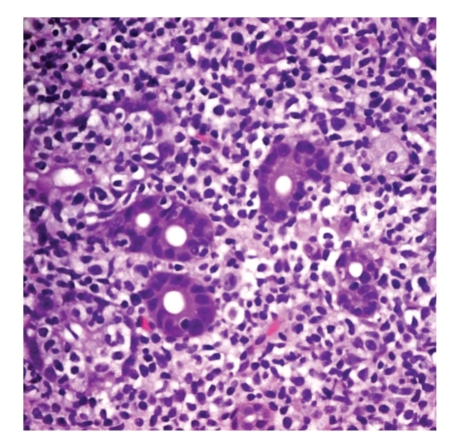
Lymphoepithelial lesions. This infiltration and destruction of the gastric gland epithelium by lymphocytes is a characteristic, but not specific, feature of MALT lymphoma (400x original magnification).

Morphological features helpful in the diagnosis of MALT lymphoma include *bona fide* epithelial and mucosal injury, typified by the so-called “lymphoepithelial lesion”, a characteristic but nonspecific infiltration of epithelial structures by lymphoma cells (Figure 3) [6, 7, 52]. Care must be taken to avoid overinterpretation of such lesions, however, as they may also appear in benign settings including reactive lymphoid infiltrates and in crypts adjacent to normal Peyer's patches. Reactive germinal centers, common in the deeper mucosa in *H. pylori* gastritis, may be colonized by lymphoma cells, with destruction of the mantle zone and the appearance of so-called “naked” follicles [6, 7]. In more extensive cases, the lymphoma can create mucosal ulcers and can infiltrate the muscularis mucosae, the submucosa, and even the muscularis propria. Muscularis mucosae infiltration and disruption can be a useful clue to the diagnosis in small biopsy specimens. While no specific IHC marker is available, an overabundance of B cells is present on CD20 stain, and aberrant coexpression of CD43 by neoplastic B-cells is found in up to 50% of cases in large series [6, 53]. In cases with extensive (or nearly complete) plasmacytic differentiation, IHC for kappa and lambda light chains can be extremely useful in highlighting a restricted plasma cell population, as such cases can closely mimic the intense plasma cell infiltrate of severe *H. pylori* gastritis. The standard application of light chain IHC, however, is not particularly helpful for determining the clonality of small lymphocytes, in our experience. 

Studies of clonality can be useful in lymphoma diagnosis, but routine use of molecular studies such as heavy-chain gene rearrangement assays to aid in a determination of lymphocyte clonality in MALT lymphoma is not our practice. Clonal populations have been demonstrated in nonneoplastic *H. pylori* gastritis and, as noted earlier, equivocal cases are probably best treated conservatively as most will respond to *H. pylori* eradication [54, 55]. In addition, a significant number of MALT lymphomas may not exhibit detectable clonal *IgH* rearrangements [54, 56]. Furthermore, biopsy specimens are usually small and easily exhausted, putting the slides cut for adequate morphological and IHC analysis at a premium. Thus, the differential diagnosis of severe gastritis and incipient lymphoma is best made based on a combination of clinical information, histomorphology, and IHC. Whether pretreatment analysis of molecular cytogenetics to determine the likelihood of treatment response is useful remains a topic of debate, although some reports indicate that it can be used in equivocal cases to help determine malignancy [36]. Reported cytogenetic abnormalities in gastric MALT lymphomas are summarized in Table 1.

Once the diagnosis of MALT lymphoma has been made and *H. pylori* eradication therapy initiated, the definition of treatment failure must be considered. While the majority of cases will respond to conservative therapy, the timing of rebiopsy for assessment of response is crucial in avoiding an inappropriate judgment of failure. Complete resolution of the lymphoid infiltrate typically takes several months, and periods as long as two years to complete resolution have been reported [14]. While it is typical practice in the setting of colonic adenomas to rebiopsy for assessment of complete polyp removal after a few weeks' time to allow for mucosal healing, at least eight to twelve weeks between initiation of therapy and followup biopsy for MALT lymphoma is more prudent. Certainly, an assessment of failure is inappropriate before at least eight to twelve weeks have elapsed, as the lymphomatous infiltrate may not look substantively different in such a short time. Even at two to three months' treatment duration (some observers suggest as long as a year), only a morphologically obvious worsening of the infiltrate or endoscopically visible increase in mass or lesion size should be considered a likely failure. In addition, molecular evidence of clonality by *IgH* gene rearrangement studies may persist for years after morphological remission is achieved, apparently without affecting patient outcome [14, 57]. Molecular analysis for t(11;18)(q21;q21) may be used in unresponsive cases, although the known association of this translocation with refractoriness to *H. pylori* eradication therapy may make such an assay unnecessary at this point in the disease course. Furthermore, there have been reports of antibiotic response in cases with this translocation [51, 58]. Thus, further study is needed to determine the utility of molecular assays in determining prognosis and directing therapy for patients refractory to conservative treatment [59].

Refractory cases may require more aggressive adjuvant therapy, including systemic chemotherapy and/or radiotherapy [12, 60]. MALT lymphomas have been found to be very sensitive to such systemic treatment [61, 62]. Cases with minimal residual mucosal disease, however, may be best managed by watchful waiting [63, 64]. Rarely, but particularly in cases that have transformed to DLBCL, surgery may be necessary to deal with complications such as intractable bleeding or perforation, and there is some discussion about whether surgical intervention should be reconsidered for primary therapy [65]. In addition to the presence of certain translocations described earlier, other factors associated with refractoriness to conservative therapy are transmural infiltration and transformation to DLBCL. A small number (around 10%) of cases will relapse, but this is typically associated with *H. pylori* reinfection [60].

To summarize, while the diagnosis of gastric MALT lymphoma and its definitive differentiation from severe *H. pylori* gastritis can be difficult and are not amenable to a specific, algorithmic approach, our diagnostic methodology involves several touchstones. First, the simple presence of a lymphoplasmacytic infiltrate, with or without *H. pylori* organisms, is insufficient for a diagnosis of MALT lymphoma and, indeed, is probably best regarded as *H. pylori*-type gastritis and treated conservatively. We generally require microscopic evidence of significant mucosal disruption and injury—ideally accompanied by macroscopic features such as an endoscopically visible ulcer, mass, or thickened gastric folds—to raise any suspicion of lymphoma. In this setting, we perform an IHC panel that includes CD3, CD20, and CD43 to confirm an excess of CD20-postive B cells, possibly with the addition of kappa and lambda light chain stains if a significant plasmacytic component is suspected. Aberrant coexpression of CD43 by the B cells adds further evidential weight to the diagnosis, but is only present in up to 50% of cases. We rarely, if ever, utilize molecular assays for either clonality or specific chromosomal abnormalities, as biopsy tissue is generally sparse and the vast majority of cases will respond to *H. pylori* eradication therapy. For followup, we recommend rebiopsy after at least 12 weeks, with the expectation that the infiltrate at that point may not be markedly better, but should at least be no worse. We routinely compare follow-up biopsies to previous material and, given the known propensity for MALT lymphoma infiltrates to persist for several months to more than a year, we assiduously avoid an assignment of treatment-refractory status to a lymphoma unless it fails to appreciably improve after several months of therapy or recognizably worsens during therapy. For follow-up biopsies, we mention the status of the infiltrate in our reports, either in the diagnostic line or in a comment, such as “Stomach, biopsy: Residual MALT lymphoma, significantly improved from the prior biopsy on [date]”.

## 5. Conclusion

Surgical pathologists commonly face the diagnosis of *H. pylori* gastritis, and severe cases can be difficult to distinguish from *H. pylori*-associated MALT lymphoma, particularly in the absence of suspicious clinical features. Happily, the large majority of such lymphoma cases respond to conservative antibiotic-based therapy for *H. pylori* eradication. Several cytogenetic abnormalities, including chromosomal translocations and trisomies, have been described in MALT lymphoma, and two translocations [t(11;18)(q21;q21) and t(1;14)(p22;q32)] are associated with resistance to conservative treatment. The routine use of molecular studies, including those for clonality such as *IgH* gene rearrangement, in the diagnosis of MALT lymphoma is controversial, however, and a trial of conservative therapy is probably the best initial approach given the propensity for response to such treatment. Careful assessment of response is crucial, since lymphomas may take several months to more than a year to exhibit a complete resolution of the malignant lymphoid infiltrate. Thus, a premature declaration of treatment refractoriness should be avoided in order to prevent the inappropriate use of more aggressive adjuvant therapy.

## Figures and Tables

**Table 1:** Cytogenetic abnormalities in gastric MALT lymphomas, along with their reported frequencies, known clinical implications and available assays for their detection.

Abnormality	% Gastric MALT	Clinical implications	Available assay(s)
t(11;18)(q21;q21)	Up to 25%	Antibiotic resistance	FISH; RT-PCR; BCL10 IHC (nuclear)
t(14;18)(q32;q21)	Up to 5%	Unknown	FISH; PCR; MALT1 and BCL10 IHC (cytoplasmic/perinuclear)
t(1;14)(p22;q32)	Rare	Antibiotic resistance	BCL10 IHC (nuclear)
t(3;14)(q27;q32)	Rare	Reported in DLBCL with concurrent MALT	FISH (break-apart); BCL6 IHC (only 25%–30%)
Trisomies	5%–65%, depending on series	3q27 most common; associated with high-stage disease	FISH
